# Potential Anti-Alzheimer Agents from Guanidinyl Tryptophan Derivatives with Activities of Membrane Adhesion and Conformational Transition Inhibitions

**DOI:** 10.3390/molecules26164863

**Published:** 2021-08-11

**Authors:** Pathomwat Wongrattanakamon, Jutamas Jiaranaikulwanitch, Opa Vajragupta, Supat Jiranusornkul, Chalermpong Saenjum, Wipawadee Yooin

**Affiliations:** 1 Laboratory for Molecular Design and Simulation (LMDS), Faculty of Pharmacy, Chiang Mai University, Chiang Mai 50200, Thailand; supat.jira@cmu.ac.th; 2 Department of Pharmaceutical Sciences, Faculty of Pharmacy, Chiang Mai University, Chiang Mai 50200, Thailand; jutamas.jia@cmu.ac.th (J.J.); chalermpong.s@cmu.ac.th (C.S.); 3 Cluster of Excellence on Biodiversity-Based Economic and Society (B.BES-CMU), Chiang Mai University, Chiang Mai 50200, Thailand; 4 Office of Research Affairs, Faculty of Pharmaceutical Sciences, Chulalongkorn University, Bangkok 10330, Thailand; opa.v@chula.ac.th

**Keywords:** Aβ monomer, Alzheimer’s disease, guanidinyl tryptophan compounds, lipid bilayer, molecular dynamics simulation, TGN4

## Abstract

Guanidinyl tryptophan derivatives TGN1, TGN2, TGN3, and TGN4 were synthesized, and these compounds were shown to possess in vitro inhibitory activity for amyloid aggregation in a previous study. Nevertheless, the influence of the TGN series of compounds on the binding and permeation behaviors of an Aβ monomer to the cell membranes was not elucidated. In this study, we investigated the effect of compounds in the TGN series on the behavior of an Aβ monomer regarding its toxicity toward the bilayer lipid membrane using molecular dynamics (MD) simulation. MD simulations suggest that TGN4 is a potential agent that can interfere with the movement of the Aβ monomer into the membrane. The MM-GBSA result demonstrated that TGN4 exhibits the highest affinity to the Aβ_1–42_ monomer but has the lowest affinity to the bilayer. Moreover, TGN4 also contributes to a decrease in the binding affinity between the Aβ_1–42_ monomer and the POPC membrane. Regarding the results of the binding mode and conformational analyses, a high number of amino-acid residues were shown to provide the binding interactions between TGN4 and the Aβ_1–42_ monomer. TGN4 also reduces the conformational transition of the Aβ_1–42_ monomer by means of interacting with the monomer. The present study presents molecular-level insights into how the TGN series of compounds affect the membrane adsorption and the conformational transition of the Aβ_1–42_ monomer, which could be valuable for the further development of new anti-Alzheimer agents.

## 1. Introduction

Alzheimer’s disease (AD) is a progressive degenerative brain disease and the most common cause of dementia [1,2]. Regarding the pathological features of AD, the disease is characterized by the accumulation of neurofibrillary tangles and senile plaques inside and outside brain cells, respectively. In these plaques, amyloid-β protein (Aβ) aggregations are observed in a variety of types. The major enzymes, β- and γ-secretases, sequentially cleavage the amyloid precursor protein into the two most common isoforms of Aβ: Aβ_40_ and Aβ_42_. Aβ_42_ is the most toxic amyloid and is selected as the target in many pharmacological studies [3]. An imbalance in its production and clearance could result in the excessive accumulation of Aβ_42_ outside the neural cells of AD patients. Furthermore, the disruption of the cell membrane may result in the internalization of Aβ into the cytosol. The in vitro study by Mrdenovic et al. [4] showed that the aggregation of the Aβ monomers can result in the formation of Aβ oligomers, which is the neurotoxic form. This neurotoxic protein is able to permeate the cell membrane. They used electrochemical impedance spectroscopy, atomic force microscopy, and MD simulation to study the effect of a fluorene-based active molecule, K162, on both Aβ aggregation and Aβ oligomer toxicity toward the lipid bilayer membrane. The results showed that the bilayer membrane integrity was preserved (by inhibiting the cell membrane permeation induced by the Aβ oligomers) when the compound was presented. Moreover, only shallow defects on the membrane surface were formed. The study shows the importance of the inhibition of Aβ aggregation, which further hinders the formation of toxic Aβ oligomers. The formation of a molecular interaction between Aβ and the membrane surface is a conceptually physiological mechanism due to the amphipathic properties of the amyloid protein and lipid structures. Charged-charged interactions between the Aβ side-chains and the lipid headgroups of the membrane are mostly electrostatic interactions that are responsible for the initial binding on the membrane surface. Hydrophobic interactions between the protein and the phospholipid acyl chains are further responsible for the accumulation of Aβ on the surface, which leads to an increased facilitation of the aggregation process. Then, the transformation of amyloidogenic proteins into fibrils can be induced via the process of misfolding and ordering provided by the membrane. The fibrils can keep certain affinities on the surface and, thus, insert and penetrate into the cytosol [5]. The initial binding could be the crucial mechanism for the design of new anti-Alzheimer drugs to obstruct the binding of Aβ to cell membranes and consequently reduce Aβ aggregation. Furthermore, there have been many previous studies regarding the conformational transition mechanism. These studies showed that the modification from the initial structures of an α-helix or random coil to a β-sheet is significant. In consequence, the inhibition of the Aβ monomer’s conformational transition behaviors may lead to a decrease in the ability of the Aβ monomer to bind to cell membranes.

In a previous study, guanidinyl tryptophan derivatives of the TGN series (Table 1) were designed. The tryptophan moiety of their molecules contributes to central nervous system penetration. The synthetic process of these compounds is as follows: compound (*S*,*Z*)-*tert*-butyl(6-((1*H*-indol-3-yl)methyl)-10,10-dimethyl-5,8-dioxo-9-oxa-2-thia-4,7-diazaundecan-3-ylidene)carbamate (T3), NH_2_-side chain (four different groups), HATU reagent, and *N*-methylmorpholine (NMM) in *N*,*N*-dimethylformamide (DMF) interact to yield the compounds *N*-*tert*-butoxycarbonyl (Boc)-TGN1 to Boc-TGN4. These compounds are further processed, and the residue is then purified to yield finally the TGN series of compounds: TGN1, TGN2, TGN3, and TGN4. In the previous study, these compounds were tested and exposed to in vitro amyloid aggregation inhibitory activity, supporting their ability to inhibit the β-amyloid cascade. In addition, the same study also examined the direct binding effect of the TGN series of compounds on Aβ and found that the binding energy value was not consistent with the anti-Aβ aggregation activity [6]. Nevertheless, the influence of the TGN series of compounds on the binding behaviors of an Aβ monomer to the cell membranes has not been elucidated. In a crowded environment, proteins normally coexist with membranes, affecting not only protein folding and aggregation but also the interactions of proteins or protein aggregates with membranes. Using lipid membrane models in computational studies could mimic a cellular environment that is more similar to the crowded condition [7].

Regarding the molecular basis for amyloid-mediated membrane damage and toxicity, it has been shown that Aβ becomes structured when it binds to a membrane surface, and that its intermediate plays a key role in membrane damage and toxicity. A major membrane damage model has been proposed: membrane destabilization via a carpet model. However, several factors have been suggested to come into play. First, electrostatic forces are supposed to lead to an effective migration of Aβ from the aqueous region to the membrane surface. Second, the association of Aβ with the membrane surface would result in an increased local peptide concentration, and membrane-bound Aβ monomeric units would interact with one another in two-dimensional space. The misfolding of the monomers results in aggregation via the formation of β-sheet rich protofibrils. The Aβ monomer aggregates may bind lipid membrane surfaces, and then the clustering of proteins on the membrane surface would induce their assembly into β sheet-rich aggregates. The peptides may also assemble to form a pore, thus enabling membrane leakage. Moreover, amyloid aggregates may grow at the membrane surface and induce lipid extraction via a detergent-like mechanism [8]. Therefore, it is important to consider the membrane aggregation-inhibitory and protofibril-disruptive capability of small molecules via the mechanisms of membrane adhesion and Aβ monomer conformational transition inhibitions due to the Aβ binding of these small molecules. Several studies have demonstrated that small molecules can protect membranes from damage. The preincubation of Aβ_42_ with a small molecule such as EGCG has been shown to weaken the membrane disruption [7].

Computational techniques are useful in helping to determine the schematic representation of the binding interaction of Aβ and other molecules. In this study, protein-ligand-membrane models consisting of (1) the Aβ_1–42_ monomer, (2) guanidinyl tryptophan ligands (TGN1, TGN2, TGN3, and TGN4), and (3) a hydrated POPC membrane bilayer were constructed. Then, the membrane adhesion behaviors of the Aβ_1–42_ monomer were investigated using all-atom explicit-water molecular dynamics (MD) simulations. This computational approach was used to clarify (1) the affinity of the guanidinyl tryptophan compounds to the Aβ_1–42_ monomer and the membrane, as well as the affinity of the Aβ_1–42_ monomer to the membrane, representing the potency of these ligands in membrane adhesion inhibition, (2) initial and post-MD simulation conformations of the Aβ_1–42_ monomers from the ligand-monomer models compared to only the monomer model, (3) the stability of the Aβ_1–42_ monomer and the ligands on the membrane surface, (4) interaction patterns which allow the binding of the ligands to the Aβ_1–42_ monomer, and (5) a diffusion parameter of the ligands, indicating their penetration into the bilayer. Free binding energies between the Aβ_1–42_ monomer and membrane, between the ligand and Aβ_1–42_ monomer, and between the ligand and membrane, indicating the affinities, were measured. The root-mean-square deviation (RMSD), indicating the stability of the Aβ_1–42_ monomer and the ligands, was calculated. A binding mode analysis elucidating interaction patterns and including hotspot amino-acid residues was performed. Additionally, the mean square displacement (MSD) was also calculated to determine ligand diffusion and stability on the membrane surface. Generally, as a promising lead compound, the selected ligand should (1) exhibit a favorable binding energy to the protein, resulting in a stable protein structure indicated by a low and stable RMSD pattern of the protein, subsequently decreasing binding affinity between the protein and membrane, (2) be stable in its binding to the protein, indicated by exhibiting a small RMSD for the ligand structure, and (3) be unfavorable to be absorbed through the membrane, shown by a lower negative binding energy to the membrane and a low MSD in the *z*-axis.

The results of the present study provide a rational basis for understanding the influence of guanidinyl tryptophan compounds on the membrane surface binding of the Aβ_1–42_ monomer, which can explain the anti-amyloid aggregation effect. This useful information can be used for the further development of potent AD drugs.

## 2. Results and Discussion

### 2.1. Adsorption of the Aβ_1–42_ Monomers on the Lipid Bilayer

**Figure 1:**
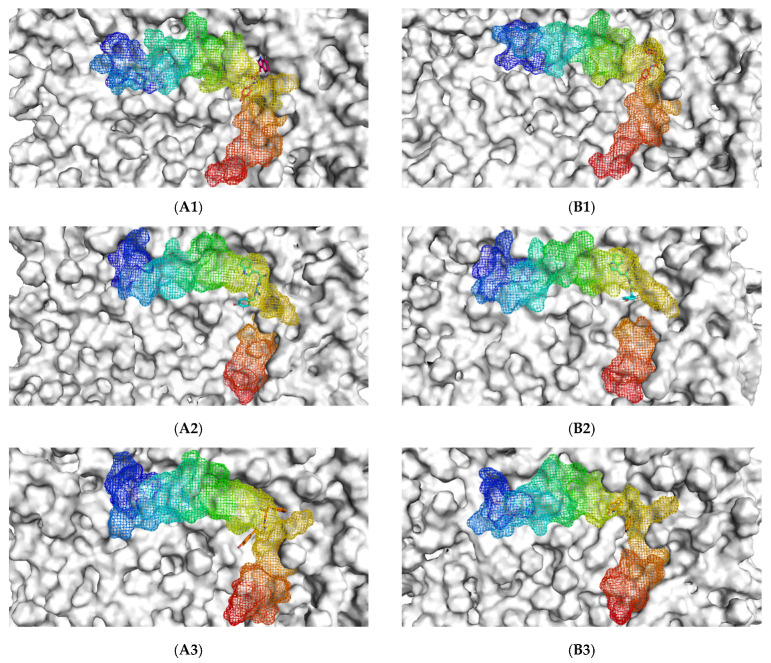

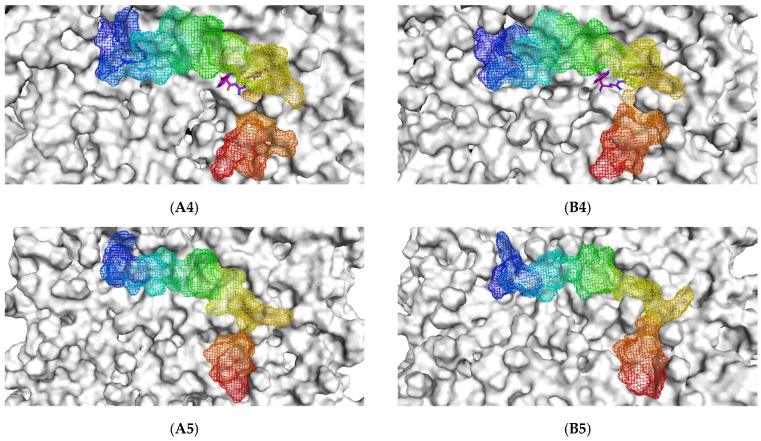
The initial binding mode retrieved from post-equilibration (**A**) and post-200 ns MD simulation binding modes (**B**) of the Aβ_1–42_ monomer (blue to red from N-terminus to C-terminus) and ligands (**1**) TGN1, (**2**) TGN2, (**3**) TGN3, and (**4**) TGN4, as well as (**5**) no ligand (only Aβ_1–42_ monomer) on the upper-layer surface of the POPC membrane (white).

The initial binding mode of the Aβ_1–42_ monomer and ligands and their accommodation at the water-lipid interface was analyzed from the post-equilibrium phase. The membrane was used in this study following previous findings that, in the exofacial leaflet of the neuronal synaptic membrane, an enrichment of POPC was observed and, moreover, the fibrillar Aβ structure exhibited higher affinity to zwitterionic membranes than to negatively charged membranes [9]. The MD simulations showed a similar initial orientation of the Aβ_1–42_ monomers on the POPC membranes of each complex (Figure 1(A1–A5)). When considering the post-200 ns MD simulation binding mode compared to the initial binding mode, it was found that the Aβ_1–42_ monomer tended to be more adsorbed into the membrane. With the exception of TGN4, the membrane that previously partially covered the C-terminal was exposed, and the C-terminal was elevated.

**Figure 2:**
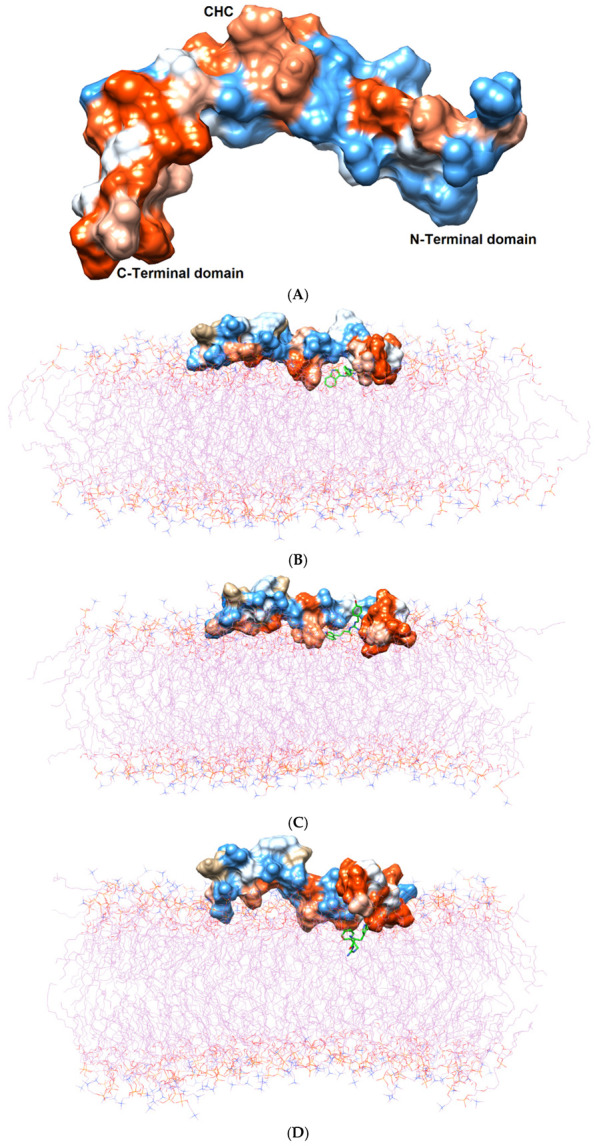

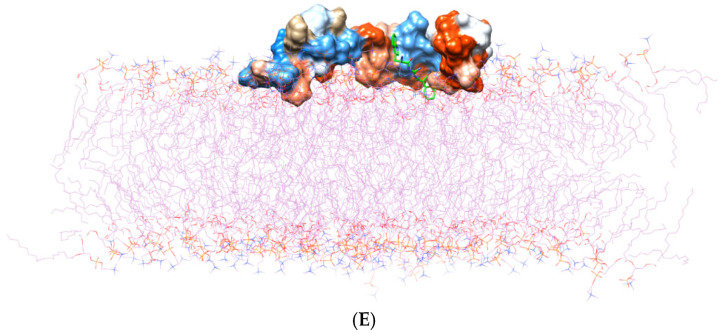
(**A**) Three domains of the Aβ_1–42_ monomer: the hydrophilic N-terminal domain, the middle domain (called the central hydrophobic cluster (CHC)), and the hydrophobic C-terminal domain. The Aβ_1–42_ monomer is colored in surface representation according to the hydrophobicity of the surface, from blue for the most hydrophilic, to white for zero, and to orange for the most hydrophobic. The post-MD simulation binding positions of (**B**) TGN1, (**C**) TGN2, (**D**) TGN3, and (**E**) TGN4 on the Aβ_1–42_ monomer surfaces and on/in the POPC bilayer (wire representation) are shown. Ligands are shown in green.

Initially, all parts of the Aβ_1–42_ monomer (Figure 1) attached to the membrane surface. The hydrophilic N-terminal domain attached to the polar heads, and the middle domains (central hydrophobic cluster) consisted of five consecutive amino-acid residues: Leu17, Val18, Phe19, Phe20, and Ala21 [10]. The hydrophobic C-terminal domain likely made close contacts with the lipid nonpolar tails. It has been shown that an amyloid fibril can expose its CHC and subsequently interact with a lipid bilayer, before being faced with conformational conversion at the water-hydrophilic head interface [11]. Regarding all ligand-Aβ_1–42_ monomer complexes, there were slight changes in the post-MD simulation positions of the ligands TGN1, TGN2, TGN3, and TGN4 and the binding Aβ_1–42_ monomers compared to their pre-MD simulation positions observed throughout the 200,000 ps trajectories. The superimposition of the pre- and post-MD simulation snapshots of each complex showed stable binding between the bilayer and Aβ_1–42_ monomer (Figure 2). The Aβ_1–42_ monomers were observed to remain on the POPC surfaces throughout 200,000 ps. The stable and well-preserved Aβ_1–42_ monomer could be a nucleating seed facilitating the aggregation of the other Aβ_1–42_ monomers. Via subsequent peptide elongation and lateral association, the monomers finally became longer and thicker fibrils [12]. For this reason, obstructing the binding of the Aβ_1–42_ monomer on the membrane surface is crucial. The modifications of the adsorption affinity and behaviors of the Aβ_1–42_ monomers on the POPC membranes by the compounds compared to the control were further analyzed.

### 2.2. Conformational Dynamics of the Aβ_1–42_ Monomer at the Water-Lipid Interface

**Figure 3:**
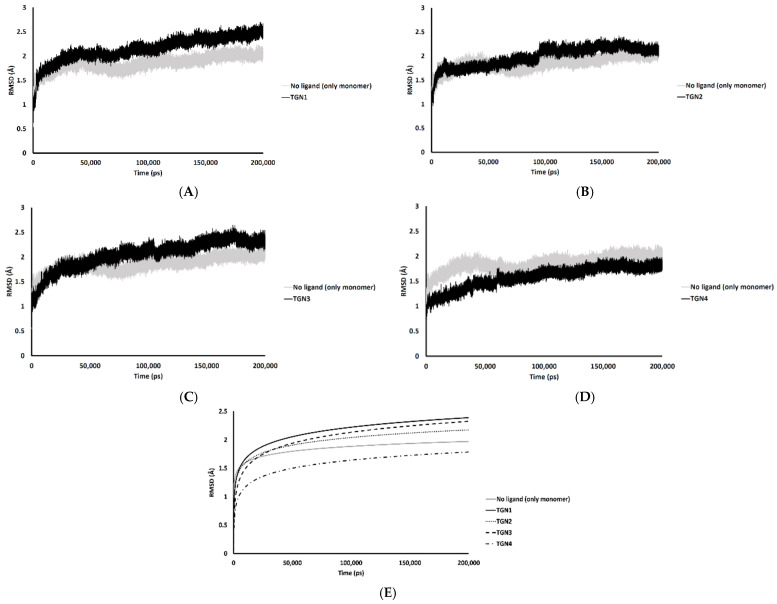
RMSD plots throughout the 200,000 ps simulation of the Aβ_1–42_ monomer coordinates from the (**A**) TGN1, (**B**) TGN2, (**C**) TGN3, and (**D**) TGN4‒Aβ_1–42_ monomer‒POPC bilayer models compared to the Aβ_1–42_ monomer‒POPC bilayer model. (**E**) The logarithmic nonlinearity of RMSD on the ps timescale of the Aβ_1–42_ monomer coordinates from the ligand‒Aβ_1–42_ monomer‒POPC bilayer models and the Aβ_1–42_ monomer‒POPC bilayer model.

**Figure 4:**
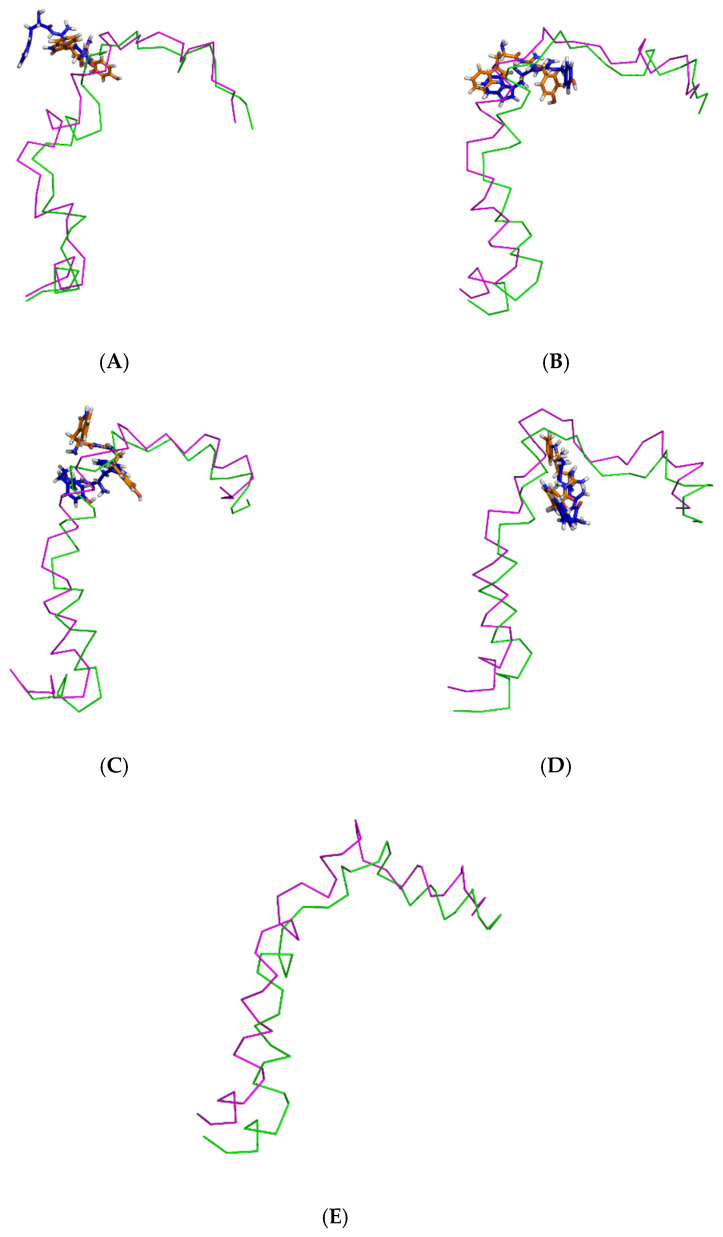
Superimposition of the pre- and post-MD simulation structures of the Aβ_1–42_ monomer shown in ribbon representation (magenta and green, respectively) and the pre- and post-MD simulation structures of each ligand shown as a stick representation (orange and blue color, respectively). The systems include ligands (**A**) TGN1, (**B**) TGN2, (**C**) TGN3, and (**D**) TGN4, as well as (**E**) no ligand (only the Aβ_1–42_ monomer).

In the 200,000 ps simulation of the TGN2 model, the Aβ_1–42_ monomer remained quite stable. Figure 3 shows the RMSD plots of the Aβ_1–42_ monomer structures from all ligand models compared to the no ligand (only monomer) model (negative control) throughout the 200,000 ps of simulations. Figure 4 shows the pre- and post-MD simulation structures/positions of Aβ_1–42_ and the ligands. The result indicated that the MD simulation of the TGN2-Aβ_1–42_ monomer complex showed a stable structure of the monomer after 100,000 ps, which verified the convergent behavior of the complex. Its RMSDs were somewhat trivially higher than the control. Regarding the TGN4 model, the monomer structure showed low structural rearrangement, which may have been due to the increase in rigidity in residue motion by the binding of TGN4 to the monomer during the simulation. The increase in its structural rigidity could be a consequence of the establishment of extensive binding interactions. However, in the TGN1 and TGN3 models, the structures of Aβ_1–42_ were found to exhibit increased RMSDs over 200,000 ps. This instability could have been due to the high motion of the ligands when they were penetrating through the bilayer, at which point they faced a steric clash with side chains of the POPC molecules. In order to substantiate this supposition, analyses of the binding affinity, RMSD, and MSD of the ligands and the RMSD of the membrane were then performed.

### 2.3. Binding Affinity Analysis

MM-GBSA calculation is a useful method for measuring the binding energy of a compound-POPC bilayer [13]. Therefore, this determination method was also employed to measure the protein-membrane and compound-protein binding energies in this study, revealing that the adsorption of the Aβ_1–42_ monomer on the membrane surface occurred by means of forming hydrophobic and electrostatic interactions, which was confirmed by the result of Yu et al. [12]. Theoretically, the MM-PBSA method for solving the Poisson-Boltzmann equation is more accurate than the MM-GBSA method. Nevertheless, the MM-GBSA calculation has pragmatically gained popularity for its satisfactory speed of calculation and comparable or even better accuracy compared to the MM-PBSA calculation in some cases [14]. In this study, the use of explicit water molecules in the MM-GBSA calculation (including the MM-PBSA calculation) may have significantly improved the accuracy of the models. The contribution of the explicit water molecules residing inside or around the binding pocket can be considered in the MM-GBSA and MM-PBSA calculations with explicit solvation models [14]. Entropy calculation using normal mode analysis was not considered due to the high computational cost of the calculation, worse performance, and reduced improvement in the accuracy of MM-GBSA and MM-PBSA in most cases for minimized structures [15,16]. Regarding the results of this study, all calculated binding energies are shown in Table 2. The calculated ligand-monomer binding energies followed the same trend as the RMSDs of the ligand-monomer complexes. TGN4 showed the highest affinity to the Aβ_1–42_ monomer (−17.8 kcal/mol of binding free energy), leading to the most stable conformation of the monomer (the lowest RMSDs). TGN2 also showed high affinity to the monomer (−14.7 kcal/mol of the energy), leading to the highly stable conformation of the Aβ_1–42_ monomer. Nevertheless, the less stable Aβ_1–42_ structures in the TGN1 and TGN3 models (high RMSDs) also reflected the low affinity to Aβ_1–42_ of TGN1 and TGN3 (−0.7 and −9.2 kcal/mol for the MM-GBSA values, respectively). Moreover, the MM-GBSA analysis showed that the monomer structures in the models of TGN1, TGN3, and TGN4 may not bind stably with the POPC membranes, with estimated binding energies of −85.9, −89.0, and −34.6 kcal/mol, respectively, which were less negative than the negative control no ligand (only monomer) model (−109.3 kcal/mol). This suggests that the existence of TGN1, TGN3, and TGN4 obviously affected the ability of the Aβ_1–42_ monomer when interacting with the membrane, as opposed to the existence of TGN2, which contributed to the binding of the monomer to the POPC bilayer and for which a highly negative value of the MM-GBSA energy was observed (−129.8 kcal/mol). As compared to the monomer-membrane binding energies, the MM-GBSA energies revealed that, in the TGN2 and TGN4 models, the ligands showed more favorable binding to the Aβ_1–42_ monomers than to the POPC membrane. On the other hand, in the TGN1 and TGN3 models, the opposite trends were observed; TGN1 and TGN3 exhibited less favorable binding affinity to the Aβ_1–42_ monomers but had highly favorable affinity to the POPC molecules. The MM-GBSA energies for TGN4 showed favorable binding to only the Aβ_1–42_ monomer (not the membrane) of this compound. Therefore, TGN4 could be classified as a promising anti-Alzheimer agent. The high stability of this ligand, as well as the low stability of the other ligands, was also further confirmed by the analyses of the ligand RMSD and MSD.

### 2.4. Conformational Dynamics and Movement of the Ligands into the POPC Bilayer

**Figure 5:**
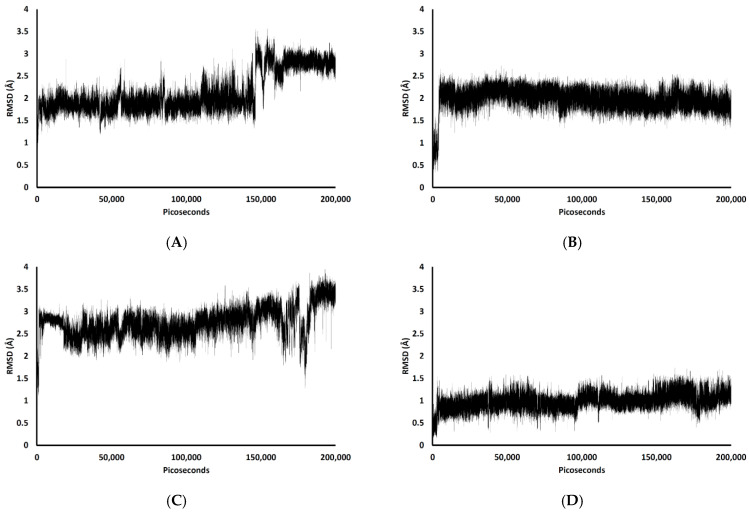
RMSD plots during the 200,000 ps simulation of the ligand coordinates from the (**A**) TGN1, (**B**) TGN2, (**C**) TGN3, and (**D**) TGN4‒Aβ_1–42_ monomer‒POPC bilayer models.

**Figure 6:**
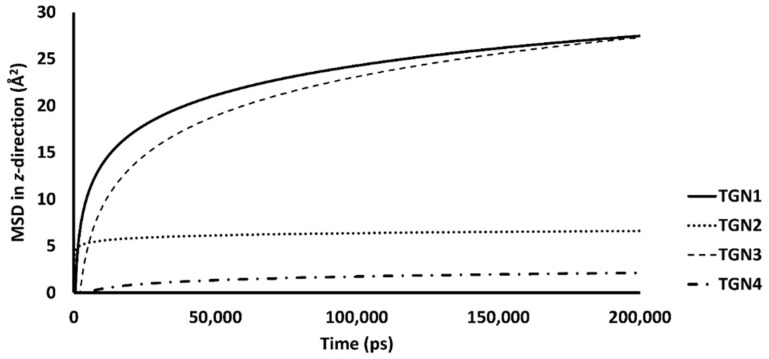
The mobilities in the *z*-direction of TGN1, TGN2, TGN3, and TGN4 in the POPC membranes are shown as the logarithmic nonlinearity of MSD on the ps timescale.

Figure 5 shows the RMSDs with respect to the initial structures of all ligands. The RMSDs of TGN2 and TGN4 stabilized after 4000 and 3000 ps respectively, and steady-state dynamics are shown. On the contrary, RMSDs of TGN1 and TGN2 fitted on the Aβ_1–42_ monomer showed fluctuation during the late phase of the 200,000 ps trajectories. The MSD patterns of all ligands (Figure 6) supportively clarified a plausible cause for the reduced stability of TGN1 and TGN3, which could have been due to the passive absorption of these compounds through the POPC lipid bilayer. Moreover, the MM-GBSA result was also confirmed by concordance with the average MSD values of the ligands. A value of *R^2^* ≈ 0.8 is shown in Figure S2 (Supplementary Materials). This suggests that the MM-GBSA analysis was capable of and suitable for measuring the affinity between the ligands and Aβ_1–42_.

**Figure 7:**
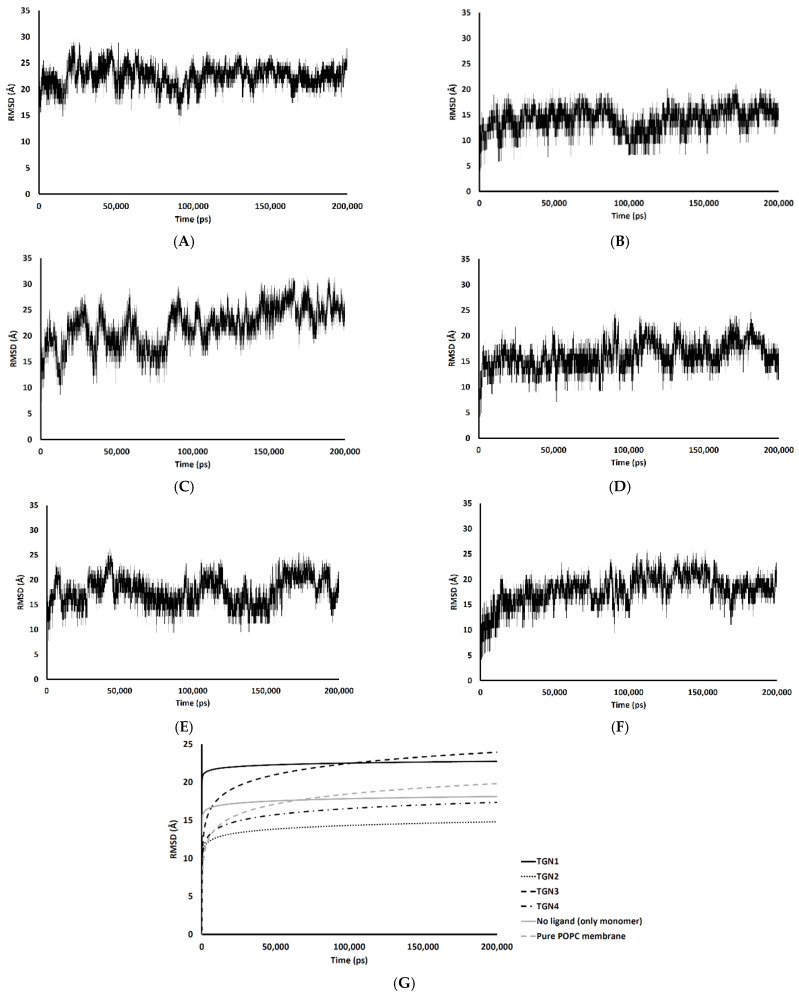
RMSD values of the membrane coordinates of (**A**) TGN1, (**B**) TGN2, (**C**) TGN3, (**D**) TGN4, (**E**) no ligand (only monomer), and (**F**) pure POPC membrane models; (**G**) the logarithmic nonlinearity of RMSD on the ps timescale of all models during the 200,000 ps simulations.

The passive diffusion of TGN1 and TGN3 into the POPC bilayer model via a transcellular pathway was primarily shown by their MSD plots as a function of time, whereas TGN2 and TGN4 showed slight motion over a small number of initial frames and stable positions throughout the latter frames. The presence of TGN1 and TGN3 at the water-lipid interface was expected to interact with some lipid residues in the bilayer and induce their mobility, which could have subsequently affected the membrane structure via a steric clash with the side chains of the POPC molecules, as shown by the RMSD plots of the POPC molecules from all models (Figure 7). The time series (in ps) of RMSDs with respect to an initial structure of the bilayer components from every model were calculated to estimate their stability. Common differences in the RMSD values among the ligand-Aβ_1–42_ monomer-POPC bilayer models, the Aβ_1–42_ monomer-POPC bilayer model, and the pure POPC bilayer model were observed.

An increase in the mobility of bilayer lipid molecules is usually caused by an interaction and steric clash involving compound penetration into a biological membrane, resulting in the high RMSDs (low stability) observed [17]. In the TGN1 and TGN3 models, the higher RMSDs than that of the Aβ_1–42_ monomer-POPC bilayer model and the pure POPC bilayer model showed that the diffusion processes of TGN1 and TGN3 increased the chain movement of the surrounding lipid molecules. On the contrary, the RMSDs of the TGN4 model were not different compared to the Aβ_1–42_ monomer-POPC bilayer model and the pure POPC bilayer model, showing that there was no insertion of the ligand into the POPC bilayer when the ligand interacted with the lipid molecules with a low binding affinity to the membrane, which was due to the tight binding with the Aβ_1–42_ monomer, as shown by the MM-GBSA analysis. The RMSDs of the TGN2 model were appreciably lower than those of the Aβ_1–42_ monomer-POPC bilayer model and the pure POPC bilayer model, showing an increase in the rigidity of the membrane that may have been due to the high binding affinity of the Aβ_1–42_ monomer to the POPC molecules compared to the no ligand model, contributed by the binding of TGN2.

### 2.5. Binding Modes of the Post-MD Simulation Structures of the Ligand-Aβ_1–42_ Monomer Complexes

**Figure 8:**
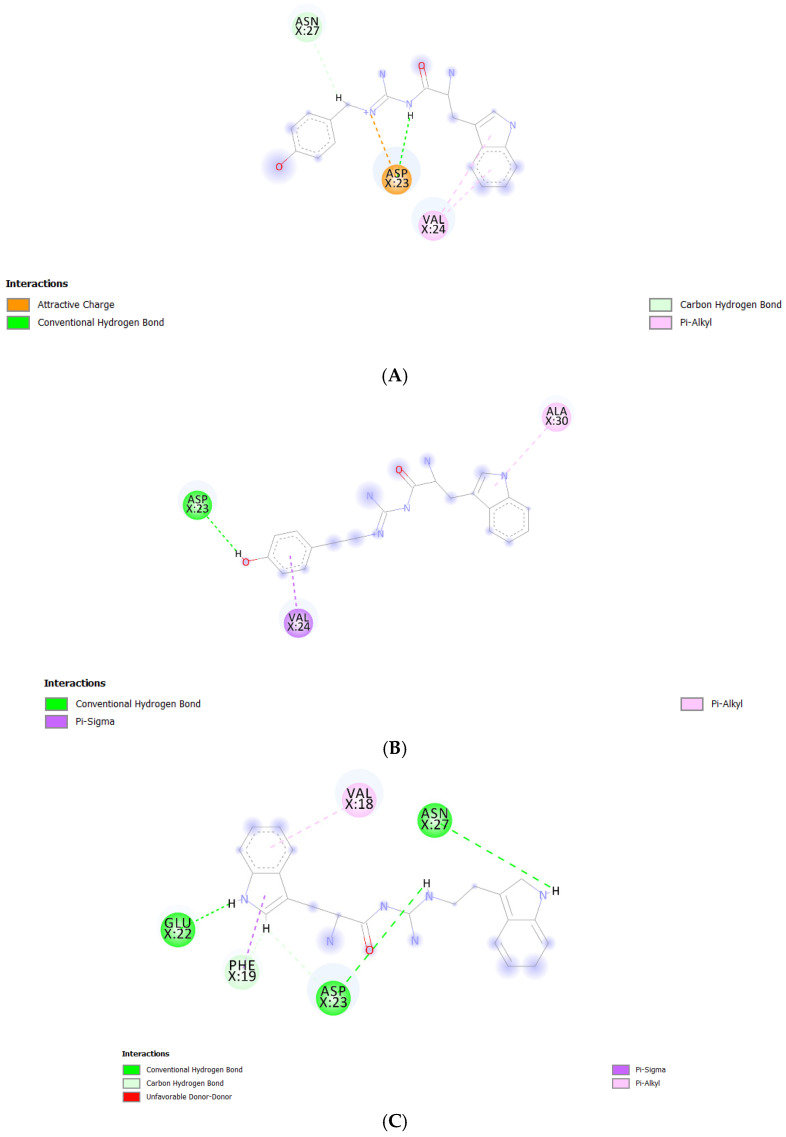
Post-MD simulation binding mode analysis of (**A**) TGN2–, (**B**) TGN3–, and (**C**) TGN4‒Aβ_1–42_ monomer complexes.

We performed further binding mode analysis using Biovia Discovery Studio Visualizer 2020 to provide a rationale toward the binding affinity levels—from low to high—of the Aβ_1–42_ monomers to the POPC bilayers from all models. The binding modes of all compounds except TGN1 are shown in Figure 8. The compound ranking based on MM-GBSA energy values was in the order of TGN4 > TGN2 > TGN3 > TGN1. In the binding of TGN4 to the Aβ_1–42_ monomer, this ligand occupied the highest number of amino acids (five residues) and formed six interactions. This occupancy may have produced the highest binding free energy to the Aβ_1–42_ monomer of TGN4, which was also supported by the very weak binding affinity of TGN4 to the POPC molecules, as shown in Table 1. TGN2 and TGN3 occupied the same number of amino acids (three residues). However, the binding of TGN2 to the Aβ_1–42_ monomer produced five binding interactions—more than TGN3 (only three interactions). The occupancy pattern of TGN2 provides a rationale for the second-ranked binding free energy to the Aβ_1–42_ monomer of TGN2, which is also influenced by the fact that TGN2 had quite a strong binding affinity to the POPC molecules, but still weaker than the binding affinity to the monomer. This result suggests that TGN2 could be a linker molecule as a consequence of exhibiting this binding pattern. On the contrary, TGN3 had a lower binding affinity to the monomer than the binding affinity to the POPC molecules. Regarding the binding of TGN1 to the Aβ_1–42_ monomer, this ligand exhibited a very low binding affinity to the monomer, whereas it had a very high binding affinity to the POPC molecules. No amino acid occupied by TGN1 was detected. For this reason, both TGN1 and TGN3 were able to smoothly insert their structures into the upper leaflets of the POPC bilayers and then penetrate into the bilayer. The amino-acid residues occupied by all ligands are shown in Table 3.

According to the results, initially, the CHC likely made close contacts with the lipid nonpolar tail. The blockage of this part could have resulted in a decrease in binding affinity to the lipid bilayer. The binding mode analysis could also provide a rationale for the reduction in binding affinity of the Aβ_1–42_ monomer to the POPC membrane. TGN4 could directly bind to CHC at Val18 and Phe19, as well as reduce the conformational transition of the native conformation; thus, it was not suitable for the C-terminal domain to lie on the membrane surface by forming a V-shape conformation, as shown in Figure 2E and Figure 4D, whereas TGN2 and TGN3 could only form interactions with the hydrophobic (Val24, Ala30), and polar (Asn27) and charged (Asp23) amino-acid residues near to the CHC. While TGN3 inserted its structure into the membrane during the simulation, the ring moieties of TGN3 still attached to the monomer surface at the monomer-membrane interface, as shown in Figure 2D; thus, its binding modes could be detected afterward. These modes could result in a smaller reduction in the monomer-membrane binding affinity than that exhibited by TGN4.

In the analysis, the electrostatic interactions between the charged residues—which were Glu22, Asp23, and Lys28—and the POPC polar groups were identified as the major driving forces. They could contribute to the insertion and penetration of the hydrophobic C-terminal domain through the membrane polar region. The penetration could result in the further loss of the native structure of the Aβ monomer and also cause a local membrane thinning effect [18]. Therefore, the occupation of Glu22 by TGN4 and of Asp23 by TGN3 and TGN4 may be beneficial for the cell membrane protection caused by Aβ.

**Figure 9:**
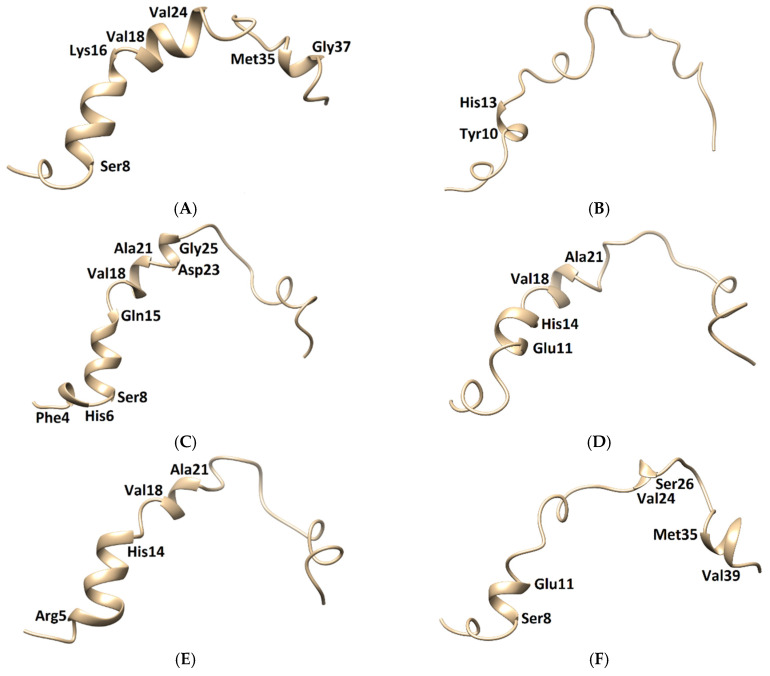
Three-dimensional structures of the Aβ_1–42_ monomers. (**A**) The NMR structure, with Model 3 and the other models taken in the last frames from (**B**) TGN1, (**C**) TGN2, (**D**) TGN3, (**E**) TGN4, and (**F**) no ligand (only monomer) models, showing the conformational transformed structures.

Another study by Narang et al. [19] provided the same result as that obtained by this study. CHC (also including the adjacent regions) was a key binding site with which the ligands favorably interacted. They examined the compound bis-tryptoline triazole (BTT) synthesized by Jiaranaikulwanitch et al. [20] for its binding interaction to the Aβ_1–42_ monomer using molecular docking and MD simulations. The complexed docking was analyzed, and the occupied residues in CHC were Lys16, Val18, and Phe19, which play a key role in the aggregation of the Aβ_1–42_ monomer [10]. Moreover, the study of Liu et al. also showed a similar result; by analyzing the binding modes of edaravone, the same residues (Lys16, Val18, and Glu22) were identified to significantly contribute to edaravone-monomer binding. It was shown that the side chain of Phe19 provided a nonpolar interaction, the main chains of Phe19 and Val24 provided electrostatic interactions, and the side chains of Glu22 and Asp23 (charged residues) provided electrostatic interactions [21]. This finding is confirmed by the result of the present study; with regard to the binding of TGN2, Asp23 formed an attractive charge interaction and a conventional hydrogen bond, while Val24 formed two pi-alkyl interactions with the ligand. Regarding the binding of TGN3, Asp23 formed a conventional hydrogen bond, and Val24 formed a pi-sigma interaction with the ligand. Regarding the binding of TGN4, Phe19 formed a pi-sigma interaction, Glu22 formed a conventional hydrogen bond, and Asp23 formed conventional and carbon hydrogen bonds with the ligand. The blockage of the CHC residues could not only result in the inhibition of the Aβ_1–42_ monomer to the membrane, but also reduce the conformational transition and the subsequent aggregation of the Aβ_1–42_ monomer, as shown in Figure 9.

The membrane-spanning monomer structures from all systems were observed to have slightly penetrated inside the membrane before becoming stable, as shown by their MSD patterns in the *z*-direction in Figure S3 (Supplementary Materials). These structures could insert their hydrophobic tails into the membrane and afterward form a strand-turn-strand cluster from Leu17 and Val36. Then, oligomers with tetrameric and hexameric β-sheet subunits could be formed by these stable monomers. These could trigger membrane damage and subsequent cellular toxicity [22]. The MD simulation elucidated the inhibitory mechanism of the guanidinyl tryptophan compounds against the aggregation of the Aβ_1–42_ monomer on the membrane surface. It also provided information regarding the reduction of the conformational transition to the aggregation-prone β-sheet of the N-terminal and CHC native helical coils. The MM-GBSA and structural analysis results clarified the ligand-monomer binding with high affinity, which could hinder the transitional process of the monomer. The binding of TGN4 obviously stabilized the native helical coils of the Aβ_1–42_ monomer as compared to the no ligand (only monomer) model, as shown in Figure 9 and Table 4. Although the binding of TGN2 conserved the native helical coils, due to the induction of the binding affinity between the Aβ_1–42_ monomer and the POPC membrane in the TGN2 model, TGN2 could not be further considered as a candidate. In the TGN1 model, the Aβ_1–42_ monomer lost many helical coils after the MD simulation, which could have been due to the unstable interactions caused by the absorption of the ligand and the high movement of the membrane (Figure 7A) during the absorption. Moreover, the low coil structure of the Aβ_1–42_ monomer could comfortably aggregate on the membrane surface.

The molecular mechanisms of amyloid aggregation with a biological membrane have been studied using both experimental and computational methods. Those results are consistent with the present findings. For example, the study of Mrdenovic et al. [4] into the fluorene-based compound K162 and their electrochemical impedance spectroscopy results showed that cell membrane integrity was preserved when both Aβ oligomers and K162 were presented. No pore formed in the membrane, which was confirmed by atomic force microscopy imaging. The lipid bilayer protection from Aβ oligomers may be due to the binding of K162 to hydrophobic residues of Aβ aggregates. These residues are relevant for both Aβ aggregation and Aβ oligomer toxicity. Therefore, further aggregation is unfavorable. Another study was performed by Dong et al. [7], and they indicated that it was significant to consider the influences of the membrane in the study of the protofibril-disruptive capability of some molecules, including the green tea compound epigallocatechin-3-gallate (EGCG). Multiple all-atom molecular dynamic simulations were used to investigate the effect of EGCG on the Aβ_42_ protofibril in the presence of a mixed POPC/POPG (7:3) membrane bilayer. Their results showed a preference of EGCG to bind to the lipid bilayer, which could alter the binding interactions between the Aβ_42_ protofibril and the POPC/POPG membrane, resulting in a decrease in membrane thinning. This modification indicated a protective effect of EGCG on the lipid bilayer. Moreover, EGCG also exhibited a disruptive effect on Aβ_42_ protofibril by destabilizing the two hydrophobic core regions and disrupting the intrachain salt bridges.

## 3. Conclusions

In this MD simulation study, TGN4 was selected as a candidate lead compound on the basis of several findings. First, MM-GBSA analysis was used to explore the affinity of the TGN compound series to the Aβ_1–42_ monomer and the membrane, and the affinity of the Aβ_1–42_ monomer to the membrane, indicating the potency of the compounds in membrane adhesion inhibition. The result showed that TGN4 exhibited a large binding energy to the Aβ_1–42_ monomer, resulting in a rigid and stable structure (a low and stable RMSD pattern) of the monomer and subsequently decreasing the binding affinity between the monomer and the membrane. Moreover, this compound was unfavorable for adsorption on the membrane surface due to its less negative binding energy. Second, the stability of the Aβ_1–42_ monomer and TGN4 on the membrane surface was shown by measuring their RMSDs. TGN4 exhibited small RMSDs, indicating the stability of its binding on the monomer surface. The RMSD pattern of the Aβ_1–42_ monomer in the TGN4 model was also low and stable, indicating the rigidity and stability of its structure. Third, initial and post-MD simulation conformations of the Aβ_1–42_ monomers from the TGN4-monomer models compared to the only monomer model were analyzed using UCSF Chimera. The conformational transition of the Aβ_1–42_ monomer from the native helical coils to the amyloid-prone structure was shown in the no ligand (only monomer) model, which could promote aggregation. The result confirmed that the conformational transition of the Aβ_1–42_ monomer is reduced by TGN4 and some of the native helical coils are conserved, and a new coil is also generated. Fourth, interaction patterns allowed the binding of TGN4 to the Aβ_1–42_ monomer. The result showed that the binding interactions between TGN4 and the Aβ_1–42_ monomer may contribute an inhibitory effect. The MM-GBSA result indicated that the contributions are from the nonpolar and polar interactions. The binding mode analysis also showed the formed electrostatic interactions between TGN4 and the hotspot residues in the binding process. Fifth, the low MSD pattern (in the *z*-axis) indicated no penetration of TGN4 into the lipid bilayer, which also reflects its stability on the membrane surface. These findings can support the design of more potent compounds against membrane adhesion and conformational transition, leading to the subsequent aggregation of the Aβ protein.

In conclusion, TGN4 tends to disrupt the Aβ_42_ protofibril by hindering the conformational transition to the aggregation-prone β-sheet through the interactions with CHC at Val18 and Phe19 and by obstructing the binding of Aβ_42_ protofibril to the lipid bilayer. These may lead to the reduction of toxic Aβ oligomers. The present study provides insights into the molecular mechanisms of TGN4 as an inhibitor of toxic Aβ oligomers in the membrane environment.

## 4. Materials and Methods

### 4.1. Lipid Bilayer Construction

A hydrated lipid bilayer for singly monosaturated 1-palmitoyl-2-oleoyl-*sn*-glycero-3-phosphocholine (POPC) lipid was constructed employing the CHARMM-GUI Membrane Builder [23]. The bilayer model contained 238 lipid molecules (119 lipid molecules in each leaflet) in a rectangular box and 20,335 TIP3P water molecules surrounding the lipid above and below it. The system sizes of the POPC membrane model were ~90 Å × 90 Å × 122 Å (*X*, *Y*, *Z*). The bilayer height was implemented in the *z*-axis. A POPC lipid bilayer model is generally selected for many in silico studies [24], including Aβ-lipid bilayer interaction studies [9,12,25,26], due to the proximity of its dynamical parameters to the experimental results [24].

### 4.2. Ligand and Protein Preparation

The 3D structures of TGN1, TGN2, TGN3, and TGN4 synthesized from the previous study [6] were initially drawn using ChemBioDraw Ultra 13.10 and then were transformed into 3D structures using ChemBio3D Ultra 13.10 (PerkinElmer, Waltham, MA, USA). The structures were then optimized with the Gaussian 09 program (Gaussian Inc., Wallingford, CT, USA) using the B3LYP model with a 6-31G (d, *p*) basis set [27]. The finally obtained structures were used as MD simulated ligands. An Aβ_1–42_ monomer simulated structure was extracted from the NMR structures of Aβ_1–42_ in aqueous solution (PDB code: 1Z0Q) [28]. This pdb file contained 30 models and, according to its structure validation report, Model 3 was considered representative because it showed the most similarity to other models.

### 4.3. Molecular Docking

A molecular docking technique can be generally used to construct a molecule-lipid membrane binding model [29]. In this study, AutoDock with the Lamarckian genetic algorithm (LGA) was used to separately build binding models for (a) the ligands to the POPC membrane and (b) the Aβ_1–42_ monomer to the POPC membrane. The grids were created to cover the center surface area of the membrane upper leaflet with 70 × 70 × 30 Å and 126 × 126 × 30 Å spaced 0.375 Å for the ligand and Aβ_1–42_ monomer docking, respectively. Parameters for the GA runs were set to 200 for the ligand docking and 10 for the Aβ_1–42_ monomer docking. A tolerance of 1.0 Å root-mean-square deviation (RMSD) was used for docking pose clustering. The ligand and Aβ_1–42_ monomer poses showing the lowest final docked energy values (kcal/mol) were selected as candidate poses to further construct a Aβ_1–42_-ligand-POPC MD simulation model.

### 4.4. MD Simulation Protocol

The MD simulations of Aβ_1–42_-TGN ligand-lipid bilayer complexes with explicit water molecules and apo Aβ_1–42_-lipid bilayer complex with explicit water molecules were performed with AMBER18 software on graphics processing units (GPUs) via the PMEMD dynamics engine. The best conformation of the Aβ_1–42_ monomer and each TGN ligand from molecular docking were first placed on the upper layer surface of the lipid bilayer to mimic an initially adsorbed state. The force field ff03.r1 [30] was employed for the entire simulated system. Force field parameters for the guanidinyl tryptophan compounds were generated by the Antechamber module. The topology and coordinate parameters for each guanidinyl tryptophan compound-Aβ_1–42_-POPC membrane model were generated with tLeap. To achieve the physiological ionic strength in the range of 0.100–0.150 M at pH 7.4 [12], counter ions of 0.070 M KCl and 0.070 M NaCl were added to reach a total concentration of 0.140 M, and the other three Na^+^ ions were used to neutralize the system. The whole system required minimization, heating, and equilibration. In the heating stage, the temperature was increased gradually from 0 to 300 K over a period of 20 picoseconds (ps) of the NVT protocol, followed by 60 ps of NVT equilibration to obtain conformations as the initial structure for the analyses. A standard methodology for the simulated system was set following the previous study [17]. The NPT production run of 200,000 ps with 300 K and 1 atm was run to generate a dynamical model of the POPC membrane and the other corresponding molecules. The MD simulation was used to determine the the Aβ_1–42_-POPC binding parameter of each complex represented by the MM-GBSA binding free energy. The MM-GBSA methods were used to calculate the binding free energy (Δ*G*_binding_) between the Aβ_1–42_ monomer and the membrane, between the Aβ_1–42_ monomer and the ligand, and between the ligand and the membrane. The conformational entropy evaluation (normal mode analysis) requires large amounts of CPU resources. Therefore, the approximation of the calculation of the binding free energy by removing this term from the MM-GBSA equation has been widely used, including in this study, as the removal of the entropic evaluation can be considered for the analysis and comparison of structurally similar compounds [31]. All the trajectories were used for the calculation. Second, structural properties (from both initial and post-MD simulation structures) of the Aβ_1–42_ monomer-ligand complexes on the membrane surface, which included binding positions/modes, the RMSD and MSD of both Aβ_1-42_ monomer and ligands, and conformations, were analyzed. The Molecular Mechanics Generalized Born Surface Area (MM-GBSA) method with the MMPBSA.py script was used to calculate the peptide-bilayer binding energy. The MM-GBSA energy values of the 200,000 ps period were calculated from the representative 200 frames for Ligand-Membrane and protein-membrane binding energies, and 20,000 frames were used for the ligand-protein binding energy. The MSD in all *Z*-directions and the RMSD over the 200,000 ps period with respect to the initial structure were calculated by the cpptraj module. All structures were visualized by PyMOL [32] and UCSF Chimera [33].

## Figures and Tables

**Table 1:** The structure and the activity of the TGN series of compounds [6].

Compound	IUPAC Name	2D Structure	Anti-Amyloid Aggregation % Inhibition at 100 μM (±SD)
TGN1	(*S*)-2-Amino-*N*-(*N*-(4-hydroxyphenyl)carbamimidoyl)-3-(1*H*-indol-3-yl)propanamide	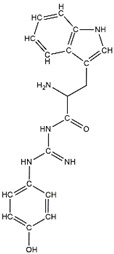	36.1 (±0.5) *
TGN2	(*S*)-2-Amino-*N*-(*N*-(4-hydroxybenzyl)carbamimidoyl)-3-(1*H*-indol-3-yl)propanamide	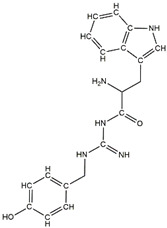	87.0 (±0.8)
TGN3	(*S*)-2-Amino-*N*-(*N*-(4-hydroxyphenethyl)carbamimidoyl)-3-(1*H*-indol-3-yl)propanamide	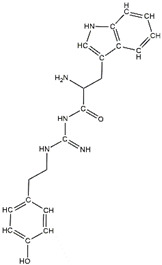	54.3 (±2.4) *
TGN4	(*S*)-*N*-(*N*-(2-(1*H*-Indol-2-yl)ethyl)carbamimidoyl)-2-amino-3-(1*H*-indol-3-yl))propanamide	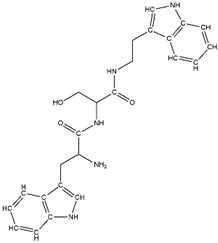	49. 8 (±1.5) *

Data are means ± SD (*n* = 3); * *p* < 0.001 compared with the positive control of each group (paired *t*-test statistic from Microsoft Excel).

**Table 2:** Interaction of the Aβ_1–42_ monomer and ligands with the POPC molecules. Molecule-bilayer binding energies (MM-GBSA) of all models were calculated over the full trajectories and are listed here.

Model	MM-GBSA Binding Energy in kcal/mol (Standard Deviation)		
	Monomer-Membrane	Ligand-Monomer	Ligand-Membrane
TGN1	−85.9 (12.2)	−0.7 (3.3382)	−28.5 (4.3)
TGN2	−129.8 (17.3)	−14.7 (2.4)	−11.3 (4.0)
TGN3	−89.0 (12.0)	−9.2 (4.2)	−12.7 (8.2)
TGN4	−34.6 (8.6)	−17.8 (2.6)	−2.9 (6.2)
No ligand (only monomer)	−109.3 (10.3)	-	-

**Table 3:** List of amino-acid residues occupied by the compounds.

Compound	Interaction and Occupied Residue	Residue of the Central Hydrophobic Cluster (CHC)
TGN1	*Undetectable*	-
TGN2	*Attractive charge interaction*: Asp23 (1 interaction) *Conventional hydrogen bond*: Asp23 (1 bond) *Carbon hydrogen bond*: Asn27 (1 bond) *Pi-Alkyl interaction*: Val24 (2 interactions)	-
TGN3	*Conventional hydrogen bond*: Asp23 (1 bond) *Pi-Alkyl interaction*: Ala30 (1 interaction) *Pi-Sigma interaction*: Val24 (1 interaction)	-
TGN4	*Conventional hydrogen bond:* Glu22 (1 bond), Asp23 (1 bond), and Asn27 (1 bond) *Carbon hydrogen bond*: Asp23 (1 bond) *Pi-Alkyl interaction*: Val18 (1 interaction) *Pi-Sigma interaction*: Phe19 (1 interaction)	Val18 and Phe19

**Table 4:** The conserved and new helical clusters of the Aβ_1–42_ monomers from all models.

Compound	Visualized Helical Cluster	Conserved Helical Cluster	New Helical Cluster
		as Compared to the Native NMR Structure	
TGN1	Tyr10–His13	*3 residues of the matched helical cluster*: Tyr10–His13	-
TGN2	Phe4–His6 Ser8–Gln15 Val18–Ala21 Asp23–Gly25	*14 residues of the matched helical cluster*: Ser8–Gln15 Val18–Ala21 Asp23–Val24	*3 residues of the new helical cluster*: Phe4–His6
TGN3	Glu11–His14 Val18–Ala21	*8 residues of the matched helical cluster*: Glu11–His14 Val18–Ala21	-
TGN4	Arg5–His14 Val18–Ala21	*11 residues of the matched helical cluster*: Ser8–His14 Val18–Ala21	*3 residues of the new helical cluster*: Arg5–Asp7
No ligand (only monomer)	Ser8–Glu11 Val24–Ser26 Met35–Val39	*7 residues of the matched helical cluster*: Ser8–Glu11 Met35–Gly37	*2 residues of the new helical cluster*: Val24–Ser26 Gly38–Val39
NMR structure	*Native helical clusters*: Ser8–Lys16 Val18–Val24 Met35–Gly37	-	-

## Data Availability

Data is available in this article and Supplementary Materials.

## Supplementary Materials

The following are available online. Figure S1. Closed views of the complexes of Aβ1-42 monomers and (A) TGN1, (B) TGN2, (C) TGN3, and (D) TGN4 on the upper-layer surfaces of the POPC membranes. The Aβ1-42 monomers, ligands, and POPC membranes are shown in magenta-mesh, orange-stick, and green-mesh representations, respectively, Figure S2. Correlation between the MM-GBSA scores and the MSD values of TGN1, TGN2, TGN3, and TGN4, Figure S3. The mobilities in the z-direction of the Aβ1-42 monomer structures in (A) TGN1, (B) TGN2, (C) TGN3, (D) TGN4, and (E) no ligand (only monomer) models in the POPC membranes are shown as MSD on the ps timescale.

## Sample Availability

Samples of the compounds are not available from the authors.
