# Supplementary material for: Potential Anti-Alzheimer Agents from Guanidinyl Tryptophan Derivatives with Activities of Membrane Adhesion and Conformational Transition Inhibitions
Source: Molecules. 2021 Aug 11;26(16):4863. doi: 10.3390/molecules26164863 (PMC8398955; doi:10.3390/molecules26164863)
Supplement: Supplementary file 1 [file molecules-26-04863-s001.zip › molecules-1290426-supplementary.pdf]

Potential Anti-Alzheimer Agents From Guanidinyl Tryptophan Derivatives with Activities of Membrane Adhesion and Conformational Transition Inhibitions

Supplementary Materials

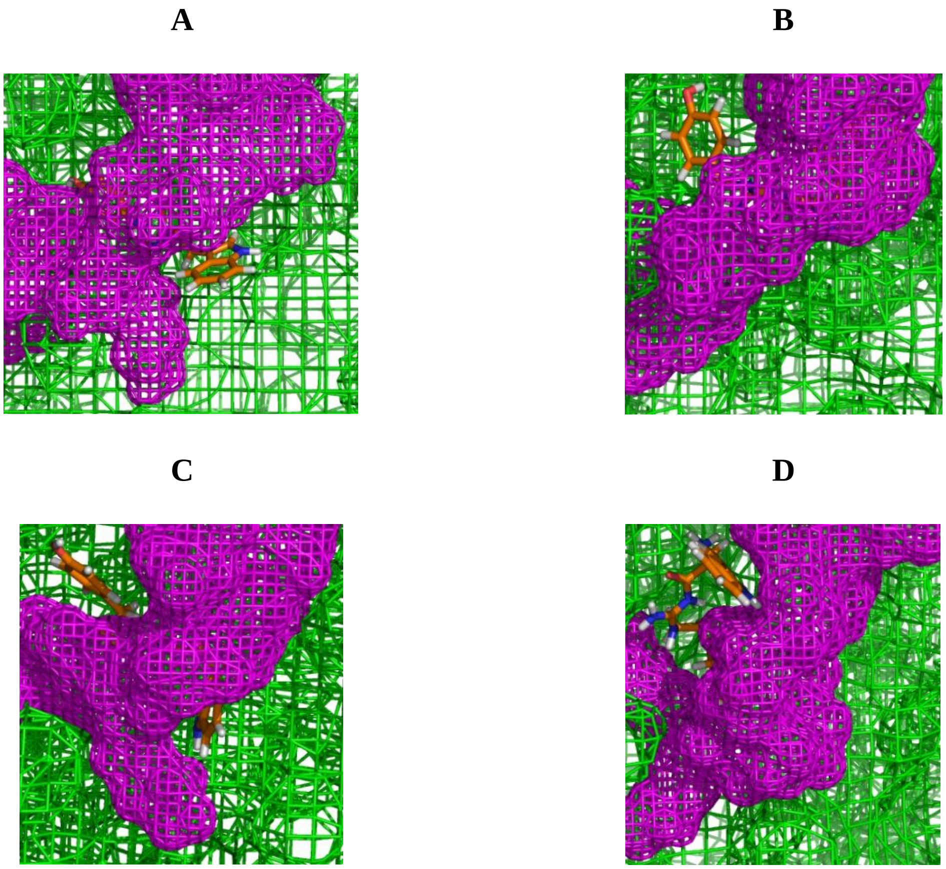

Figure S1. Closed views of the complexes of A$\beta_{1\text{-}42}$ monomers and (A) TGN1, (B) TGN2, (C) TGN3, and (D) TGN4 on the upper-layer surfaces of the POPC membranes. The A$\beta_{1\text{-}42}$ monomers, ligands, and POPC membranes are shown in magenta-mesh, orange-stick, and green-mesh representations, respectively.

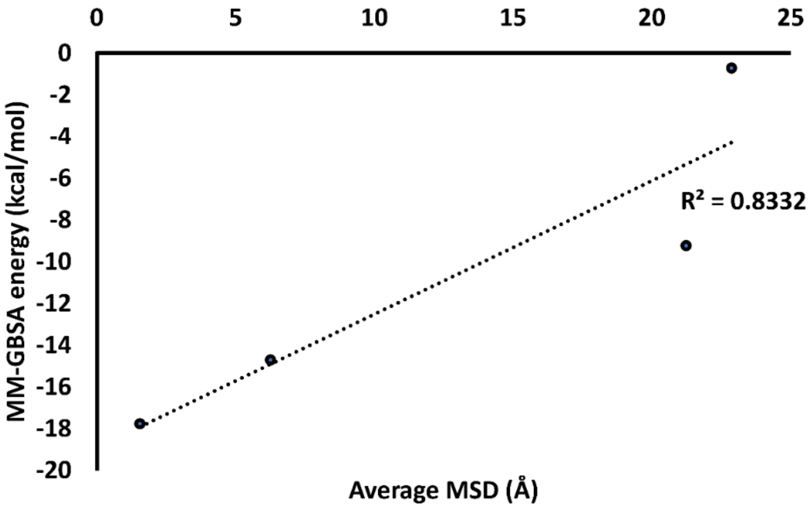

Figure S2. Correlation between the MM-GBSA scores and the MSD values of TGN1, TGN2, TGN3, and TGN4.

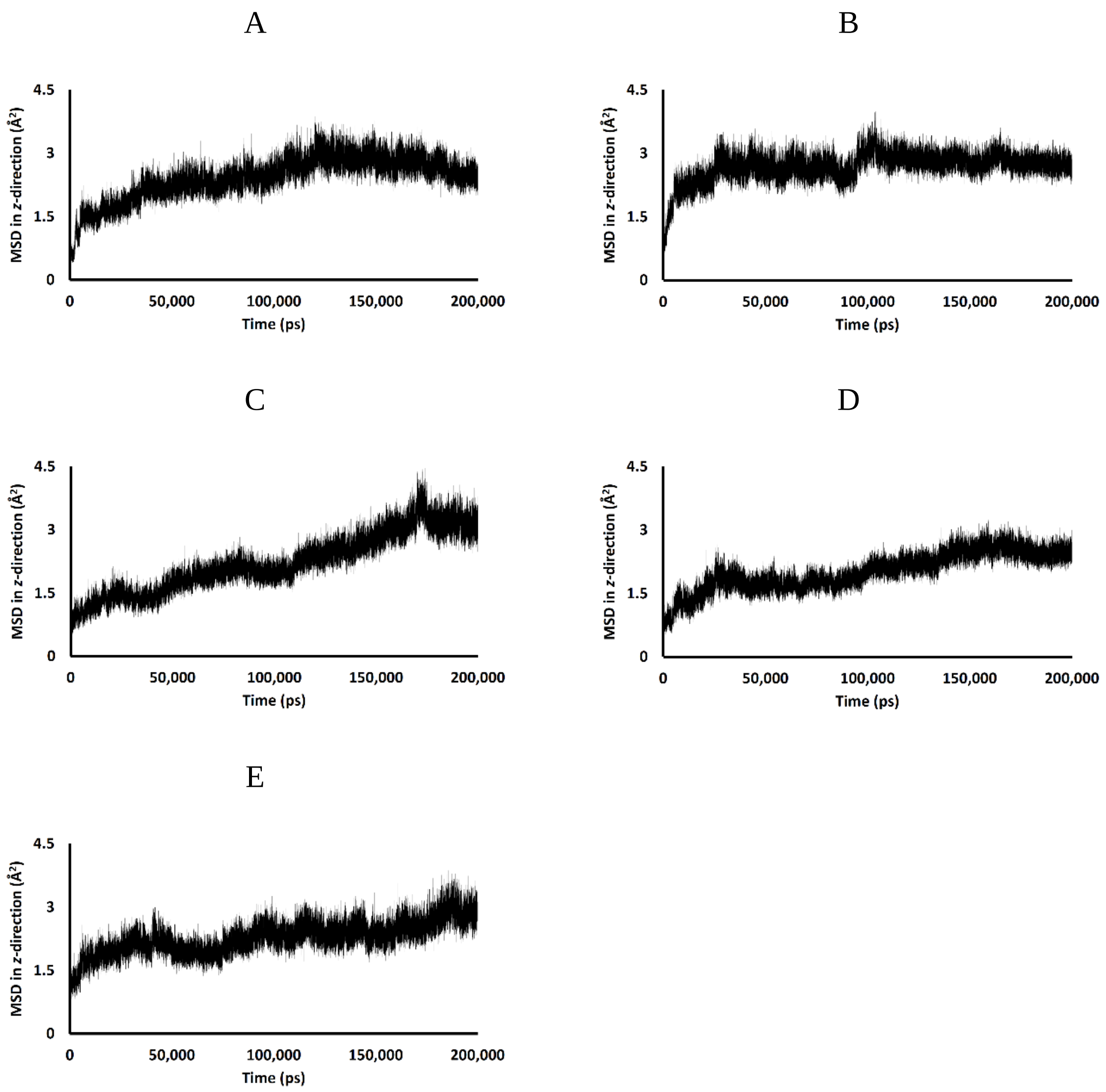

Figure S3. The mobilities in the z-direction of the A$\beta_{1\text{-}42}$ monomer structures in (A) TGN1, (B) TGN2, (C) TGN3, (D) TGN4, and (E) no ligand (only monomer) models in the POPC membranes are shown as MSD on the ps timescale.
